# Supplementary material for: Miniaturized Photo-Ionization Fourier Deconvolution Ion Mobility Spectrometer for the Detection of Volatile Organic Compounds
Source: Sensors (Basel). 2022 Jul 22;22(15):5468. doi: 10.3390/s22155468 (PMC9331717; doi:10.3390/s22155468)
Supplement: Supplementary file 1 [file sensors-22-05468-s001.zip › supplementary files.pdf]

Supporting Information

Miniaturized photo-ionization Fourier deconvolution ion mobility spectrometer for the detection of volatile organic compounds

Binwang Yang, Jianna Yu, Wen Liu, Guoxing Jing, Wenshan Li, Wenjie Liu*

College of Chemical Engineering, Xiangtan University, Xiangtan, 411105, Hunan province, China

* To whom Correspondence should be addressed.

Wenjie Liu, College of Chemical Engineering, Xiangtan University, Xiangtan, 411105, China, email: lury@sina.com. Tel: 86-136-6731-6202

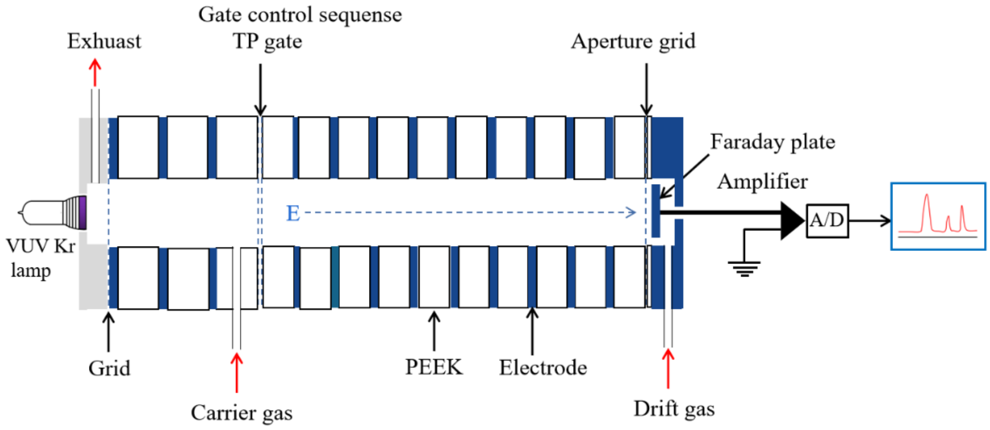

Figure S1 Schematic diagram of miniaturized Fourier deconvolution ion mobility spectrometer with a photo-ionization source

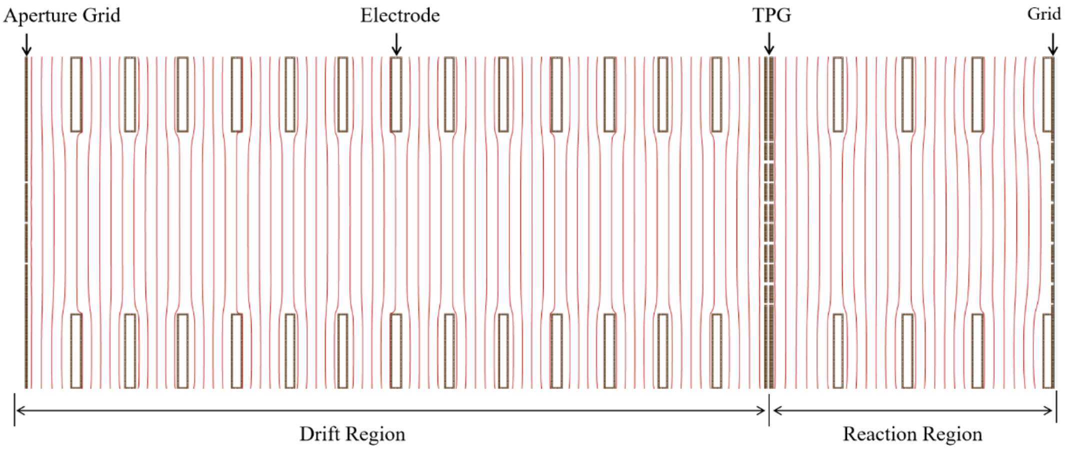

Figure S2 Electric field inside the miniaturized Fourier deconvolution ion mobility spectrometer.

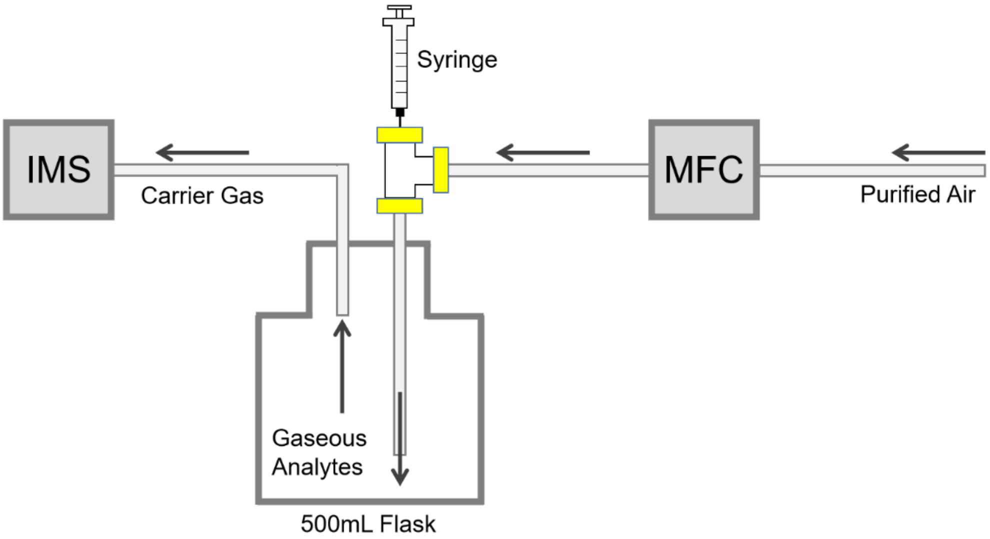

Figure S3 Schematic diagram of the exponential dilution system.

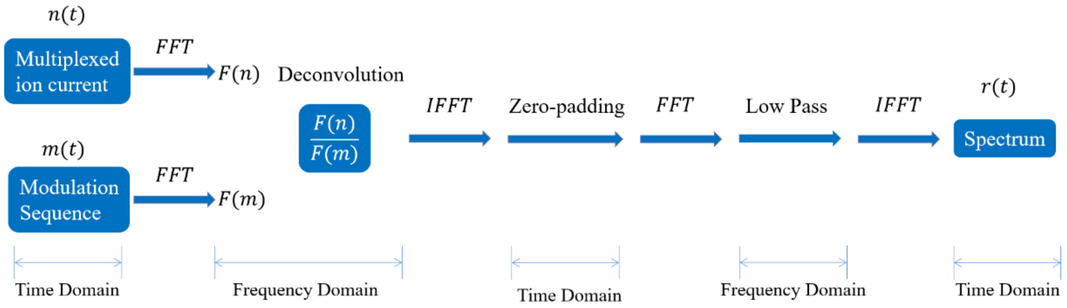

Figure S4. Systematic diagram of the data processing workflow of Fourier deconvolution ion mobility spectrometry
